# PWP1 promotes nutrient-responsive expression of 5S ribosomal RNA

**DOI:** 10.1242/bio.037911

**Published:** 2018-10-25

**Authors:** Ying Liu, Rita Cerejeira Matos, Tapio I. Heino, Ville Hietakangas

**Affiliations:** 1 Faculty of Biological and Environmental Sciences, University of Helsinki, 00790 Helsinki, Finland; 2 Institute of Biotechnology, University of Helsinki, 00790 Helsinki, Finland

**Keywords:** PWP1, NCLB, Nutrient, 5S ribosomal RNA, Transcription, RNA polymerase III

## Abstract

PWP1 is a chromatin binding protein with an important role in animal growth control downstream of mTOR-mediated nutrient sensing. PWP1 has been shown to control tissue growth by promoting the transcription of 5.8S, 18S and 28S ribosomal RNAs (rRNAs) by RNA polymerase I (Pol I). Concomitantly with Pol I, RNA Polymerase III (Pol III) contributes to ribosome biogenesis by transcribing 5S rRNA in the nucleoplasm. Pol III activity is also closely controlled by nutrient-dependent signaling, however, how the activities of Pol I and Pol III are coordinated in response to nutrient-derived signals remains insufficiently understood. Experiments in *Drosophila* larvae and human cells reported here show that PWP1 associates with the chromatin at the 5S rDNA loci and is needed for nutrient-induced expression of 5S rRNA. Similar to the Pol I target rDNAs, PWP1 epigenetically maintains 5S rDNA in a transcription competent state. Thus, as a common regulator of Pol I and Pol III, PWP1 might contribute to coordinated control of ribosomal gene expression in response to nutrition.

This article has an associated First Person interview with the first author of the paper.

## Abstract (precis)

**Summary:** We report that a chromatin-binding protein PWP1 binds to 5S ribosomal DNA, regulates its epigenetic status and controls nutrient-dependent RNA polymerase III-mediated transcription of 5S ribosomal RNA.

## INTRODUCTION

Animal growth is dynamically regulated in response to environmental factors, including nutrients. Ribosome biogenesis serves as a limiting factor for growth capacity and it is tightly controlled by nutrient-responsive signaling, including the mTOR (mechanistic target of rapamycin) pathway (Saxton and Sabatini, 2017). The ribosomal RNAs (rRNAs) are transcribed by RNA polymerases (Pols) I and III (Koš and Tollervey, 2010; Moir and Willis, 2013). Pol I acts in the nucleolus and is responsible for the expression of 5.8S, 18S and 28S rRNAs, while Pol III localizes in the nucleoplasm and transcribes 5S rRNA. Since rRNA expression involves two polymerases in different nuclear compartments, their activities need to be coordinated. mTOR signaling promotes the activities of both Pol I and III in response to nutrition, but the known downstream effectors are distinct. mTOR phosphorylates TIF-1A to activate Pol I, while in the case of Pol III regulation, mTOR phosphorylates and inhibits the repressor Maf1 (Mayer and Grummt, 2006; Rideout et al., 2012).

We have previously reported that the WD40 domain protein PWP1 contributes to nutrient-dependent control of Pol I. PWP1 is activated by mTOR through phosphorylation and elevated gene expression. PWP1 associates with the rDNA locus, maintains it in a transcription-competent epigenetic state and promotes Pol I-mediated transcription of rRNA in a nutrient-responsive manner (Liu et al., 2017). In line with the role in promoting ribosome biogenesis, *Drosophila* mutant larvae lacking PWP1 show a prominent growth retardation. In addition to the role in Pol I regulation, we observed earlier that *pwp1* mutant *Drosophila* larvae display reduced expression of RNA polymerase III targets, including 5S rRNA (Liu et al., 2017). However, it remained to be tested whether PWP1 controls Pol III-mediated transcription directly and in a nutrient-responsive manner, and whether its role in this setting is conserved in animals. Here we provide evidence that PWP1 critically contributes to nutrient-responsive expression of 5S rRNA in *Drosophila* larvae and it regulates 5S rRNA expression in a conserved manner, likely through a direct mechanism at the 5S rDNA chromatin. Our data suggest that PWP1 is a common regulator of Pol I and III and therefore has the potential to act as a coordinator of their activities.

## RESULTS

**Fig. 1.**
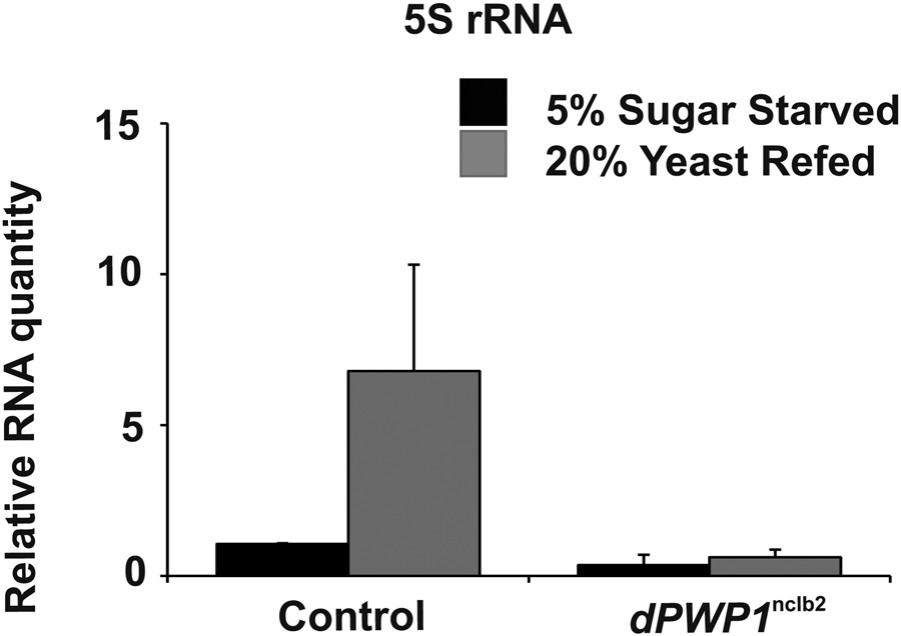
**Quantitative RT-PCR analysis of 5S rRNA expression upon high protein diet (20% yeast) re-feeding for 23 h following starvation on amino acid-deficient food (5% sucrose) of control and *pwp1*^nclb2^ null mutant larvae.** Cdk7 was used as a reference gene. *n*=5. Error bars represent standard deviation. ANOVA showed a significant genotype by feeding interaction (*F*=4.89, *P*=0.04).

Our previous study showed that PWP1 acts downstream of nutrient-responsive mTOR signaling *in vivo* in *Drosophila* and that PWP1 is essential for the induced expression of the Pol I-dependent rRNAs in response to protein-rich diet (Liu et al., 2017). As the Pol III-dependent gene expression is also dependent on nutrition (Marshall et al., 2012), we wanted to test if *Drosophila* PWP1 (dPWP1, encoded by the *nclb* gene) controls the expression of Pol III-dependent 5S rRNA in this setting. This was indeed the case, as control larvae displayed strongly elevated 5S rRNA expression upon refeeding on protein-rich yeast food following protein starvation, but this effect was blunted in *pwp1*^nclb2^ null mutant larvae (Fig. 1).

**Fig. 2.**
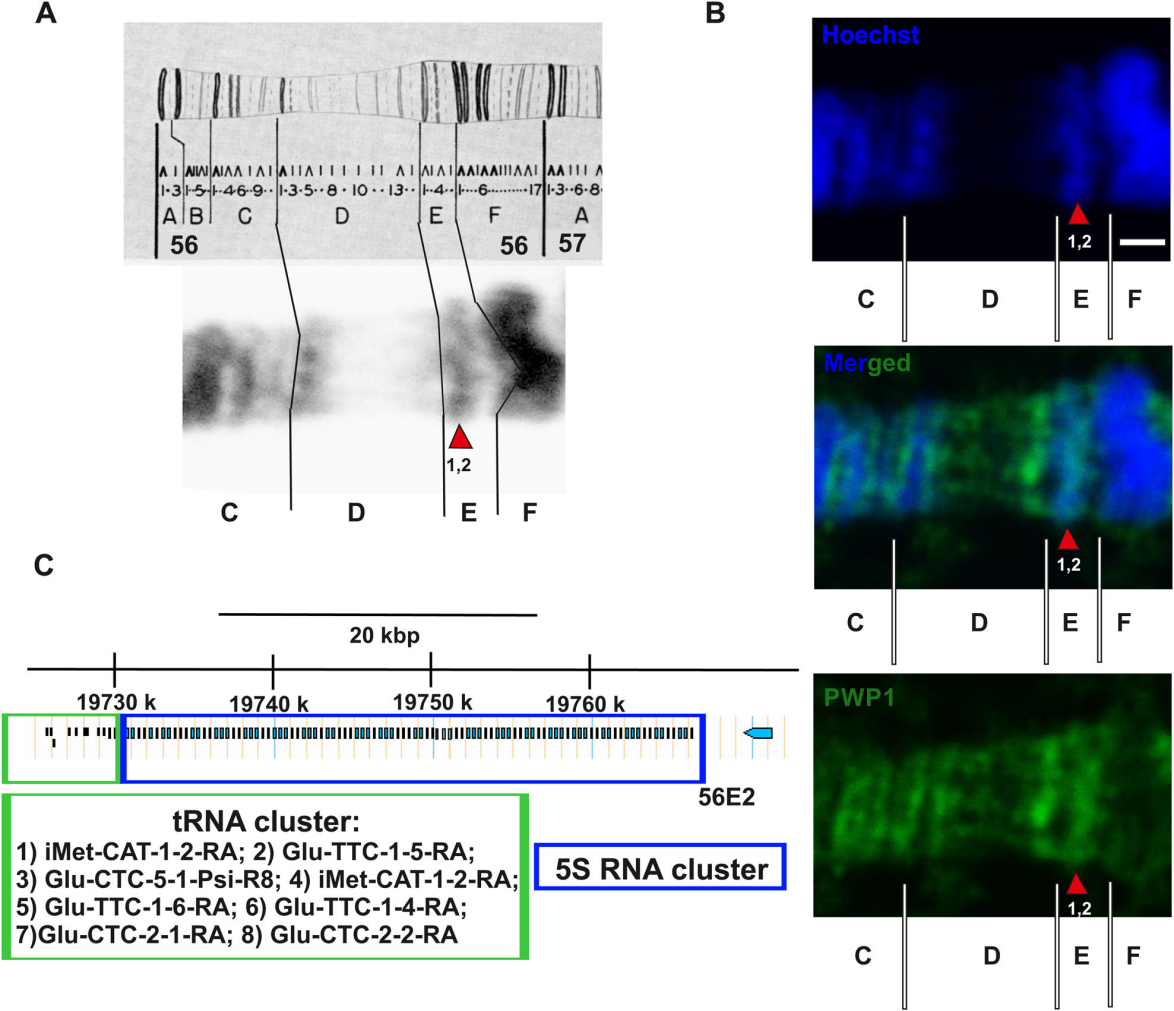
**In *Drosophila* polytene chromosomes PWP1 binds to the region 56E1-2 that contains the 5S rDNA cluster.** (A) The upper panel shows division 57 of chromosome 2R of the Bridges map (**Bridges and Bridges, 1939**). The lower figure reveals the subdivisions 57C–F in a w*^1118^* polytene chromosome. The red arrow indicates the localization of 5S locus and different tRNAs at the polytene band 56E1–2. (B) Representative images of the subdivisions 56C–F. Immunofluorescence staining with PWP1 antibody and Hoechst revealed the localization of PWP1 in the polytene band 56E1–2. Scale bar: 1 μm. (C) Scheme of the tRNA genes and tandemly repeated 5S rDNA genes as they are represented in Flybase genome browser (http://flybase.org/). The specific tRNA and 5S rDNA clusters are represented in the green and blue boxes, respectively. The blue arrow refers to the neighboring gene OR56a.

In order to analyze the possible association of endogenous dPWP1 with chromatin, we utilized immunofluorescence staining of *Drosophila* polytene chromosomes. A control without primary antibody was used to assess the specificity of the staining (Fig. S1). As previously shown (Casper et al., 2011), dPWP1 displayed a specific staining pattern, with strongest signal in weakly DAPI stained interband regions (Fig. S1). We focused our attention on the division 56E, which contains Pol III targets, including the cluster of 5S rDNA as well as several tRNA-encoding loci (Fig. 2A). Previous work has shown localization of BRF, a Pol III initiation factor subunit, into this specific region (Takada et al., 2000). Strong PWP1 staining was observed in 56E, in particular in subdivisions 56E1 and 56E2, which harbor the cluster of Pol III targets (Fig. 2B,C). In conclusion, our data implies that *Drosophila* PWP1 associates with chromatin in the vicinity of Pol III targets.

**Fig. 3.**
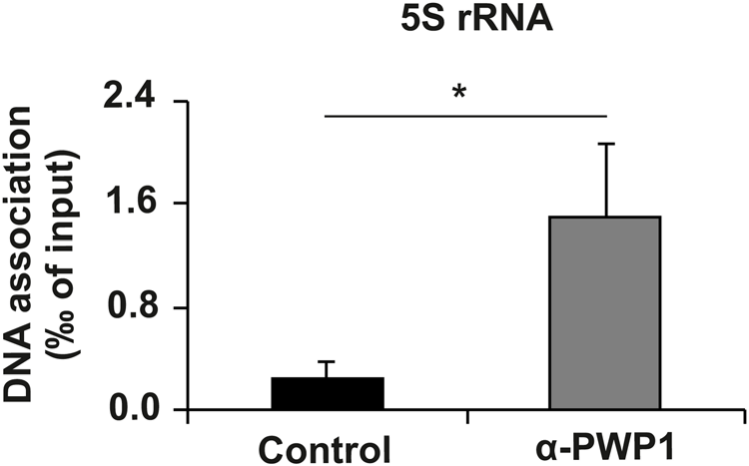
**Enrichment of PWP1 binding on 5S ribosomal RNA was revealed by chromatin immunoprecipitation (ChIP) in HeLa cells.** *n*=3. Error bars represent standard deviation. *P<0.05 (Student's *t*-test).

**Fig. 4.**
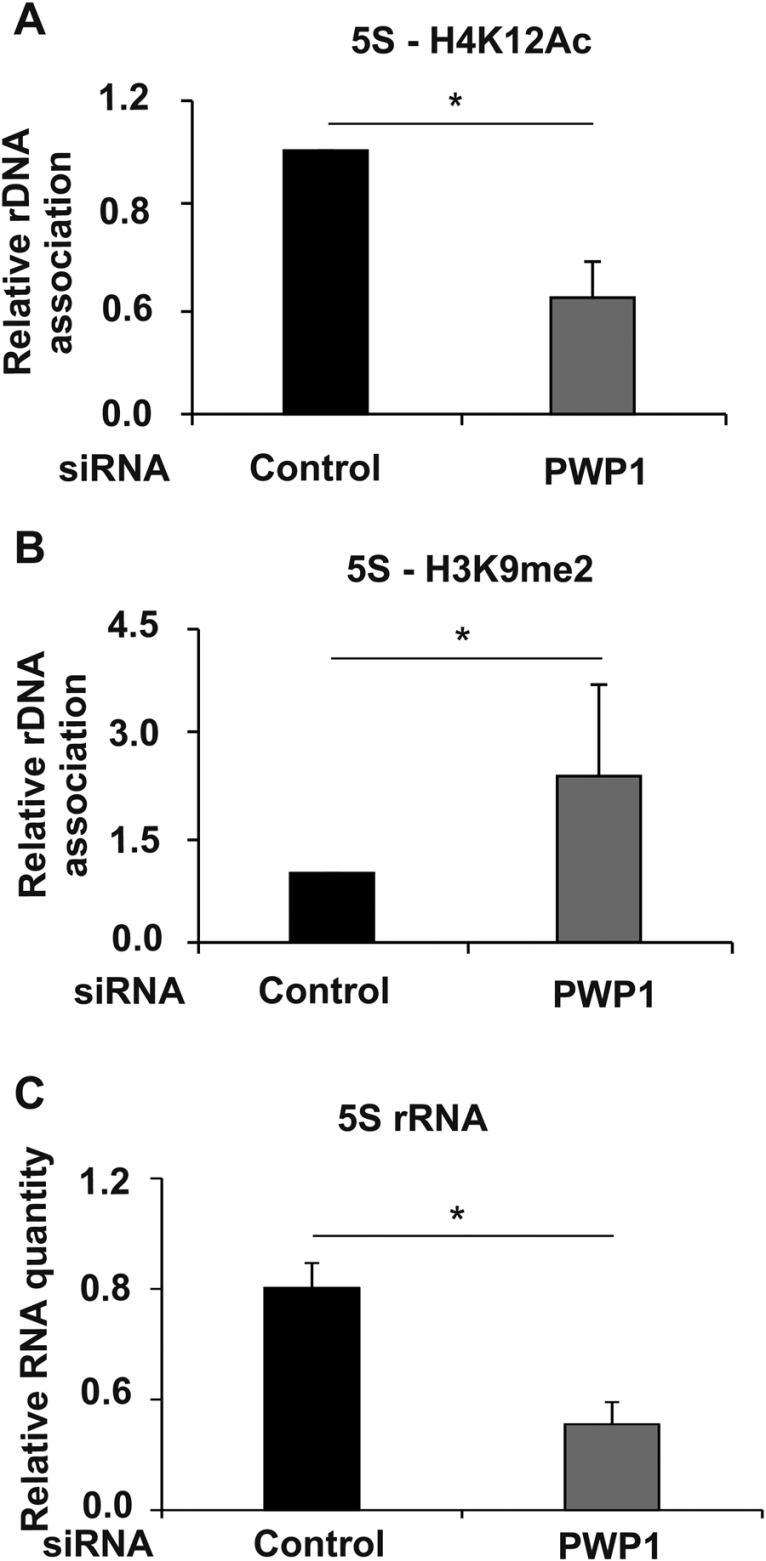
**PWP1 controls the epigenetic status of 5S rDNA.** (A) ChIP revealed reduced relative levels of H4K12Ac in the 5S rDNA region following PWP1 depletion in U2OS cells. *n*=3. Error bars display standard deviation. *P<0.05 (Student's *t*-test). (B) ChIP revealed increased relative levels of H3K9me2 on 5S rDNA following PWP1 depletion in U2OS cells. *n*=3. Error bars represent standard deviation. * *P*<0.05 (Student's *t*-test). (C) Quantitative RT-PCR analysis of 5S rRNA expression in U2OS cells following PWP1 depletion by siRNA. Human GAPDH was used as a reference gene. *n*=3. Error bars represent standard deviation. * *P*<0.05 (Student's *t*-test).

To achieve a better spatial resolution, we used chromatin immunoprecipitation (ChIP) in human cell lines. A significant enrichment of PWP1 was observed in the 5S rDNA gene region (Fig. 3). PWP1 was earlier shown to regulate the epigenetic status of Pol I target loci (Liu et al., 2017). Therefore, we wanted to analyze if similar regulation occurs in the 5S rDNA genomic region. To this end, we used knockdown of PWP1 (Fig. S2) in combination with ChIP to analyze Histone 4 lysine 12 (H4K12) acetylation, which is associated with active transcription, as well as H3K9 dimethylation, a repression-associated modification. Similar to the earlier observations on the Pol I target loci (Liu et al., 2017), knockdown of PWP1 reduced the level of H4K12 modification on 5S rDNA loci (Fig. 4A), while increasing the level of repressive H3K9 dimethylation (Fig. 4B). To confirm the functional consequences of these changes to 5S rRNA expression, we measured 5S rRNA expression in U2OS cells and observed reduced expression upon PWP1 knockdown (Fig. 4C).

## DISCUSSION

Here we show that dPWP1 is essential for nutrient-dependent expression of 5S rRNA and it associates with 5S rDNA gene region, suggesting involvement in direct Pol III regulation. The observed role for PWP1 outside the nucleolus was not surprising, as immunofluorescence analysis of PWP1 in *Drosophila* fat body shows PWP1 localization in both nucleolus and nucleoplasm (Liu et al., 2017). Furthermore, earlier findings in *Drosophila* polytene chromosomes have shown PWP1 colocalization with active Pol II (Casper et al., 2011). In the case of Pol I regulation, nucleolar recruitment of TFIIH, which promotes the elongation of Pol I, was impaired by the loss of PWP1 (Liu et al., 2017). Future studies are needed to address the mechanistic role of PWP1 in Pol III-dependent transcription and the possible similarities and differences to the Pol I regulation. We observed that PWP1 was needed to maintain high H4K12 acetylation while suppressing the H3K9 dimethylation. Little is known about the role of histone modifications on 5S rDNA, but in *Xenopus*, a high degree of histone acetylation has been correlated with active 5S rRNA expression (Bhargava, 2013). Our findings show evidence for a similar mode of regulation of histone modification in Pol I and Pol III target rDNA loci. While this study focused on the role of PWP1 in 5S rRNA expression, Pol III also transcribes hundreds of other genes, such as tRNAs, non-coding RNAs involved in splicing, gene regulation, protein targeting and mitochondrial RNA processing (Canella et al., 2010; White, 2011). Future studies are needed to comprehensively address the role of PWP1 in different types of Pol III target genes.

A particularly appealing prospect emanating from our findings is the possible role for PWP1 in coordinated control of Pol I and Pol III activities. While it remains to be tested whether mTOR-mediated phosphorylation controls PWP1 activity in Pol III, dPWP1 expression levels are under mTOR regulation, which is likely reflected in Pol III (Liu et al., 2017). A likely activator of *pwp1* expression downstream of mTOR is transcription factor Myc, an oncogene and a master regulator of ribosome biogenesis (Liu et al., 2017). Thus, PWP1 might serve as a chromatin-level coordinator of Pol I and Pol III activities downstream of Myc, which in turn integrates signals from multiple growth-regulating cues. Interestingly, human PWP1 expression is highly elevated in aggressive tumors and knockdown of PWP1 leads to inhibition of tumor cell proliferation (Liu et al., 2017). As inhibition of ribosome biogenesis is an emerging strategy for cancer therapy (Drygin et al., 2010; Hein et al., 2013), the role of PWP1 in the joint control of Pol I and Pol III makes PWP1 an appealing target.

## MATERIALS AND METHODS

### *Drosophila* stocks and maintenance

Flies were maintained either at 25°C as previous described (Havula et al., 2013), or were grown on modified food containing 0.5% (w/v) agar, 2.5% (v/v) Nipagin (methylparaben) in PBS and supplemented with 5% (w/v) sucrose or 20% (w/v) dry baker's yeast. Stocks used in this study are *w*^1118^ (BDSC 6326) and *pwp1*^nclb2^ (Casper et al., 2011).

### Cell culture and RNAi

HeLa and U2OS cells (ATCC) were cultured at 37°C in Dulbecco's modified Eagle's medium (Sigma-Aldrich) supplemented with 10% fetal bovine serum (Life Technologies), GlutaMax (Life Technologies), and penicillin/streptomycin (Life Technologies). siRNA used was SMART pool: siGENOME PWP1. The transfection of siRNA was performed using Lipofectamine RNAiMAX (Life Technologies) according to the manufacturer's protocol.

### Quantitative RT-PCR

Total RNA was extracted from *Drosophila* larvae or U2OS cells using the Nucleospin RNA II kit (Macherey-Nagel), and cDNA was synthesized using the RevertAid H Minus First Strand cDNA Synthesis Kit (Thermo Fisher Scientific) or the SensiFAST™ cDNA Synthesis Kit (Bioline) according to the manufacturer's protocol. qPCR was performed with the Light cycler480 Real-Time PCR System (Roche) using SensiFAST™ SYBR No-ROX kit (Bioline). The primers used are: human 5S rRNA (5′ CCATACCACCCTGAACGCGC 3′, 5′ AGCACCCGGTATTCCCAGGC 3′), human GAPDH (5′ TCACCACCATGGAGAAGGCT 3′, 5′ TCATACTTCTCATGGTTCACACCC 3′), human PWP1 (5’ GACAGGACGCTTGATGATGATGAGC 3′, 5′ GATCTTGATCATTACTCCCGTAGACCG 3′) *Drosophila* 5S rRNA (5′ CCATACCACGCTGAATACATCGG 3′, 5′ ACGCGGTGTTCCCAAGCG 3′), *Drosophila* CDK7 (5′ GGGTCAGTTTGCCACAGTTT 3′, 5′ GATCACCTCCAGATCCGTG 3′).

### Chromatin immunoprecipitation

Antibodies used for chromatin immunoprecipitation are anti-PWP1 (Abcam, ab190794), anti-Histone H3 (Abcam, ab1791), anti-Histone H4 (acetyl K12) (Abcam, ab46983) and anti-Histone H3 (di methyl K9) (Abcam, ab1220). The chromatin of HeLa and U2OS cells were collected as previous described (Liu et al., 2017), and the DNA was purified using the MinElute kit (Qiagen) and was afterwards subjected to qPCR.

### Polytene chromosome preparation and staining procedures

Salivary glands were dissected in PBS from wandering third instar larvae and fixed for 3 min in a mixture of 3.7% formaldehyde and 45% acetic acid. Fixed salivary glands were spread in a drop of fixative on a siliconized cover slip which was placed on top of a slide and the coverslip was pressed strongly against it. The slide was quickly submerged into liquid nitrogen, the coverslip was removed and the slide was stored in 67% glycerol in PBS. For immunostaining the preparations were washed in cold PBS 0.1% Tween, followed by 1 h blocking in 5% normal goat serum. The samples were then incubated overnight with the primary antibody anti-dPWP1 (1:500) (Casper et al., 2011) at 4°C after which the preparations were washed three times for 10 min in cold PBS 0.1% Tween and blocked for 1 h with 5% normal goat serum. After that the samples were incubated for 2 h at room temperature using the secondary antibody AlexaFluor 488 conjugated anti goat-rabbit IgG (H+L) (1:1000). After three 10 min washes in PBS 0.1% Tween, the samples were rinsed in PBS and were stained for 5 min in Hoechst 33342 (1:10000) (#62249, Thermo Fisher Scientific), washed in PBS and mounted with SHANDON, Immu-Mount (#9990402, Thermo Fisher Scientific). Images were obtained using LSM Zeiss 700 Microscope, and were then analyzed using the LSM 700 and Fiji ImageJ software.

## Supplementary Material

Supplementary information
