# Supplementary material for: PWP1 promotes nutrient-responsive expression of 5S ribosomal RNA
Source: Biol Open. 2018 Oct 25;7(11):bio037911. doi: 10.1242/bio.037911 (PMC6262851; doi:10.1242/bio.037911)
Supplement: Supplementary information [file biolopen-7-037911-s1.pdf]

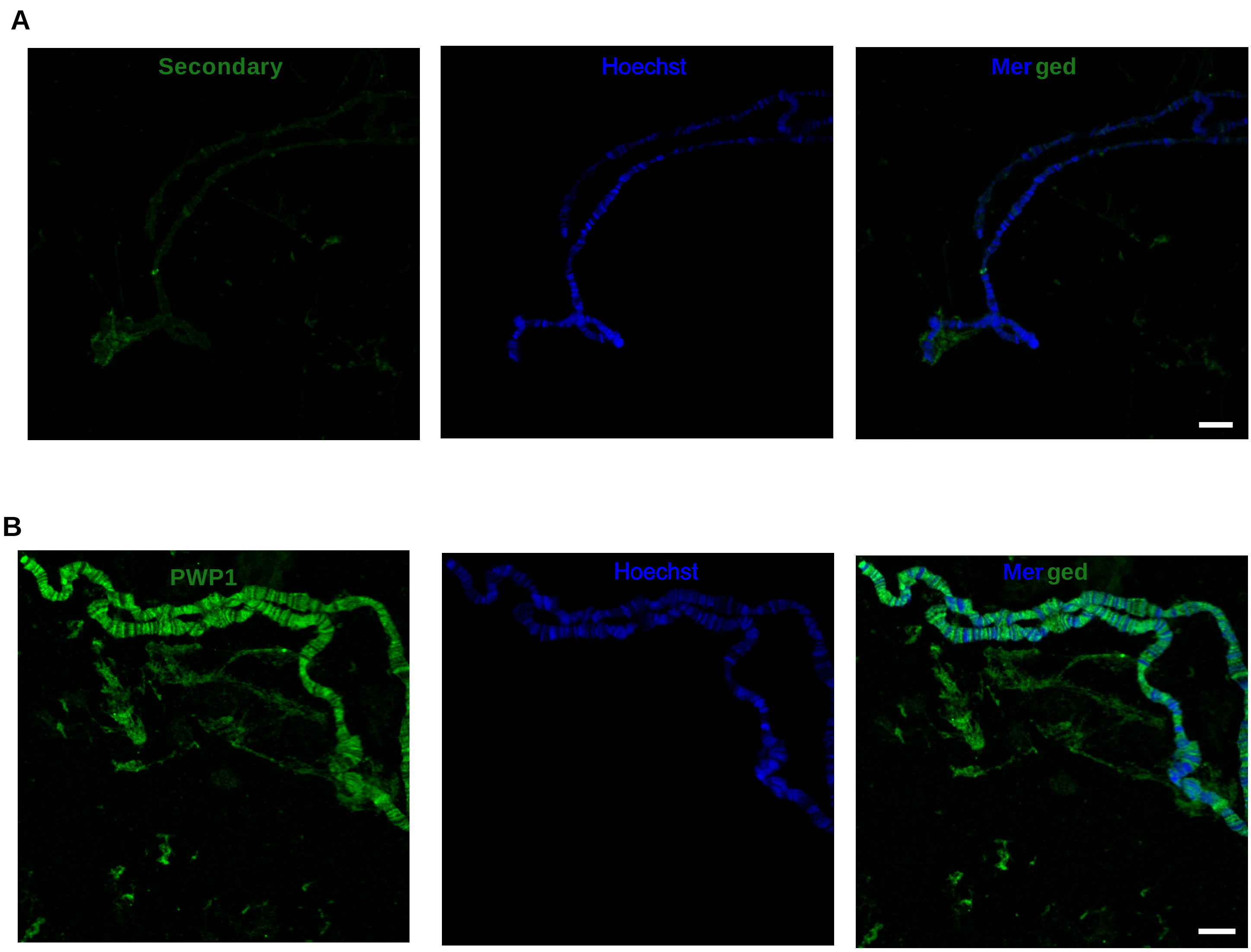

Figure S1. A) Representative image of the control polytene chromosomes stained only with the secondary antibody and Hoechst to visualize the chromatin. **B)** Immunofluorescence staining with dPWP1 antibody and Hoechst shows the specific anti-PWP1 antibody signal in multiple locations within the polytene chromosomes. Scale bar is 10 µm.

Biology Open • Supplementary information

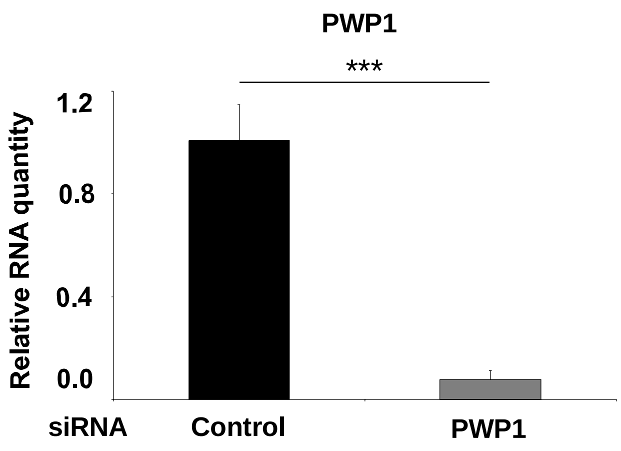

Figure S2. Quantitative RT-PCR analysis of PWP1 expression in U2OS cells following PWP1 depletion by siRNA. Human GAPDH was used as a reference gene. n=3. Error bars represent standard deviation. *** $p<0.001$ (Student's t-test).
